# ‘Wait and wait, that is the only thing they can say’: a qualitative study exploring experiences of immigrated Syrian doctors applying for medical license in Germany

**DOI:** 10.1186/s12913-020-05209-2

**Published:** 2020-04-23

**Authors:** Julika Loss, Yamen Aldoughle, Alexandra Sauter, Julia von Sommoggy

**Affiliations:** Medical Sociology, University of Regensburg, Dr.-Gessler-Straße 17, 93051 Regensburg, Germany

**Keywords:** Licensure process, Syrian doctors, Qualitative research, Migration experiences, Germany

## Abstract

**Background:**

Due to the civil war in Syria, many Syrian citizens have had to flee their country during recent years, among them many physicians. Germany is among the preferred immigration countries. Immigrant Syrian physicians could help overcome the prevailing shortage of medical specialists in Germany. This study explores the experiences and perceptions of Syrian physicians of the licensure process and job application. The study aims at understanding barriers in integrating Syrian doctors into the German health care system.

**Methods:**

We conducted 20 semi standardized interviews with Syrian doctors (*n* = 17 m; *n* = 3 f) living in different federal states in Germany. The interviews dealt with the procedure of the accreditation process, its speed and challenges, the interactions with authorities, and the job application process; they were transcribed verbatim. A detailed content analysis was performed.

**Results:**

All interviewees described the licensure process as a complex, lengthy, ever-changing and non-transparent procedure, which is perceived as a partly unfair, sometimes arbitrary bureaucracy. They often feel at mercy of Government employees and report experiences with reviewers who ask for absurd and impossible accomplishments, refuse to give information, and act at random. As a consequence, the interviewees describe themselves as depressed, irritated and/or in despair. According to the interviews, informational and practical support from official institutions was scarce. Instead, the Syrian doctors relied on peers or, in some cases, German friends to understand the requirements and seek information about the licensure process. To find a job placement, it was very helpful, if not essential, to have a German acquaintance establishing contact with possible employers. All three interviewed female doctors reported that their wearing a Hijab led to episodes of discrimination in their job search.

**Conclusions:**

The study points towards the necessity to establish an official information source which provides immigrant doctors with accurate and detailed information about the licensure process, e.g. required documents, estimated waiting times, regulation for courses and exams, criteria regarding credentials, sources of help, etc. Additionally, it seems advisable to consider providing help with regard to the job search and sensitize hospital management for cultural and religious diversity to avoid discrimination.

## Background

Since the Syrian civil war broke out in 2011, millions of Syrian citizens have fled their country and migrated to different destinations [1, 2]. The EU and especially Germany are among the preferred immigration countries. Syrians have been the largest group of asylum seekers for three consecutive years and are now the third largest group of people with a foreign nationality in Germany [3]. Due to the high educational standards in pre-war Syria, a considerable number of Syrian migrants is well qualified. In particular, the number of health care professionals, e.g. medicals doctors, is fairly high among Syrian immigrants [4, 5].

**Fig. 1:**
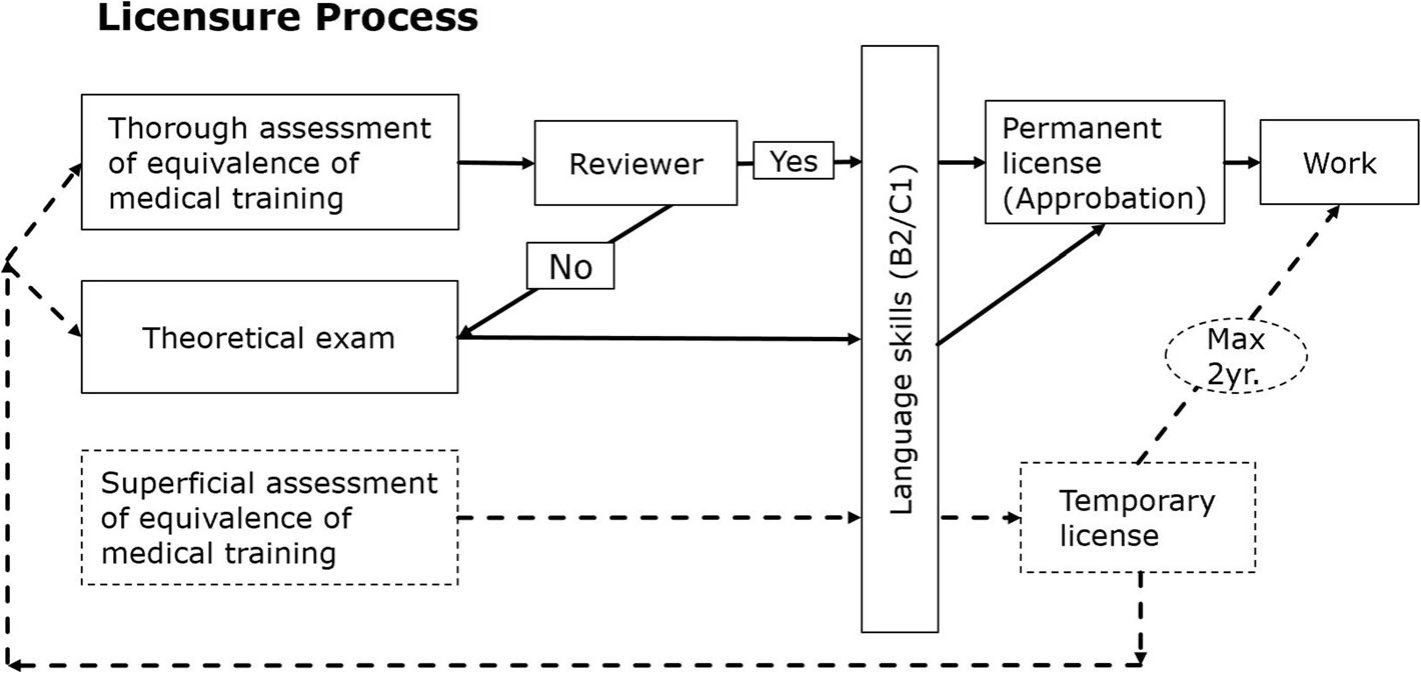
Ways to receive the permanent medical license. This figure shows alternative ways to receive the permanent medical license in Germany. Depending on the different standards in the different federal states, applicants may choose either waiting for the review of their credentials, or take a registration exam right away. As these processes may take several months, many doctors use the waiting time working on a temporary license, which is easier to obtain, but expires after 2 years. Abbreviations: yr. = years

In Germany, a relative shortage of physicians has developed over the recent years. Although absolute numbers of physicians have been rising in Germany, many vacancies cannot be filled, as the need for doctors increases every year. This can mainly be attributed to a change in working time regulations and the rising number of female doctors, who often work part-time [6–8]. Especially in the sector of primary care, in rural areas and in Eastern Germany, there is a growing lack of physicians. In addition to that, doctors educated in Germany consider emigrating to other European countries which are supposed to offer better working conditions [9, 10]. Many hospitals rely on foreign physicians to maintain health care [11, 12]. Due to the increase of migration to Germany since 2011, especially from Arabic speaking countries, the number of Arabic speaking patients is also on the rise [3]. The immigrant Syrian physicians may therefore benefit the German health care system by a) increasing the number of health care professionals available for vacant positions in hospitals, and b) helping address special (language, cultural) needs of Arabic speaking patients. The admission into the Germany medical job market, however, is difficult to obtain, especially for non-Europeans [13]. Physicians from foreign countries can start practicing with a temporary license (Berufserlaubnis) for 2 years as assisting doctor if they possess the necessary language skill (B2 or C1, depending on federal state regulations) (ref. Fig. 1) [14, 15]. In the meantime, they need to obtain a permanent license in order to continue practicing medicine. This license is called ‘Approbation’. The German medical curriculum consists of 6 years of theoretical and practical training, which is mandatory to receive a medical degree and a permanent license to practice [16]. Physicians trained in non-European countries can have their credentials assessed for equivalency to the German medical studies in a licensure process, so as to be allowed to work permanently as a doctor [17]. Records of medical training and working experience have to be handed in at the professional regulatory bodies, i.e. the State Chambers of Physicians (Landesärztekammer), where experts (‘reviewers’) compare the medical qualification of the applicant with the Germany curriculum. Another option for obtaining a permanent license is to take a theoretical exam to prove competency, either right away, or as a second option in case the medical training received in the home country is not considered equivalent [14, 17]. Furthermore, language skills are obligatory: In addition to the B2 German language level, some federal states require a language exam focusing on medical technical terms (‘Fachspracheprüfung’) [18]. The permanent license to practice medicine is necessary if an immigrant aspires to a prolonged stay or further educational training [14, 15].

Non-European (e.g. African) physicians who immigrated to European countries described the licensure process as difficult, humiliating or bureaucratic, e.g. in Austria, Belgium, or Norway [19–21]. One study analyzed experiences of immigrant doctors in Germany [17]; here, participants considered licensure processes as slow, overburdening and unfair, especially for non-EU applicants. However, the data collection was performed in 2013, before the peak of migration of Syrian doctors to Germany, which occurred in 2015 and 2016. Therefore, the interview partners were mainly from Poland, Romania, Russian federation, and Greece. Recent newspaper reports have indicated that immigrant Syrian doctors also struggle with bureaucratic difficulties in Germany, but as yet, no study has systematically explored the situation of Syrian doctors who apply for medical license in the German health care system [22]. It is therefore not clear what difficulties and barriers Syrian medical professionals may face in the admission process to the German medical labor market, and what facilitators and resources they have.

In this study, we intended to collect data on the experiences of Syrian doctors during the licensure process and the job search. Understanding the obstacles and challenges among this population can help accelerate the licensure process and make those doctors available for the healthcare system as soon as possible. Migrant physicians, therefore, play and will play an important role in securing healthcare delivery in Germany; hence, it will be indispensable to better understand how they are integrated into the German healthcare system and what challenges they face.

## Methods

The study is explorative in nature, as there are no data on experiences of Syrian physicians with the German licensure process yet. Therefore, we chose a qualitative study design. The aim is to thoroughly understand the subjective view and perspectives of people affected. Qualitative research offers a flexible process of data collection, as the researcher may probe into what the participants say and broach interesting themes further. Thus, it has a special potential to capture subjective insights that might not arise when working with predefined, standardized categories [23].

We conducted semi-structured interviews in order to remain focused on certain topics and achieve a degree of comparability. The interviewees were encouraged to make their personal view clear, and the sequence of questions was thus not strictly tied to the interview guide.

The interview guide consisted of two topics: a) Process of obtaining a permanent medical license, and b) experiences with job applications. The second topic was not discussed with those interviewees who had not yet started working or looking for a job in Germany (*n* = 5). The interview guide is detailed in Table 1.

**Table 1:** Interview Guide

#1. Process of obtaining a permanent medical license:	
✓ Please describe your licensure process in detail.	
- Which steps have you taken?	
- Which difficulties have you encountered?	
- How have you felt during the process, how was your emotional state?	
✓ How long did the whole process take?	
✓ How would you assess the speed and fairness of the process?	
✓ Please describe your interactions with the authorities / organizations which help and guide foreign doctors in the licensure process.	
#2. Job application	
✓ Please describe how you found your current job. Did an organization or agency (e.g. Jobcenter) support you?	
✓ How did you apply for your job? Can you describe differences to your home country?	
✓ How long did it take to get hired? How many applications and job interviews did it take before you found a job?	
✓ What were your experiences during the job interview?	
✓ Did you consider the application process to be fair and transparent?	

### Recruitment

Study participants had to fulfil the following criteria:

- State of origin: Syria
- completed Syrian medical degree
- German language: B2 level or better
- stay in Germany: minimum of 6 months, maximum of 48 months

Participants were recruited through a) personal contacts of a member of a research team, b) a Facebook page for Syrian doctors (الأطباء السوريين في ألمانيا = ‘*Syrian doctors in Germany*’), and c) snowball system. Thirty-five doctors contacted us with an interest to impart their experiences on professional accreditation and application processes; 20 met the inclusion criteria and consented to participate. They were interviewed subsequently [24].

### Sample

In total, 20 Syrian physicians (17 male, 3 female, 26–44 years) were interviewed. They lived in five different federal states; ten interview partners had a temporary license (Berufserlaubnis) and five a permanent license (Approbation). Five interview partners had not been granted any license yet at the point of time of the interview. Table 2 shows details of the study population. The female interview partners were IP08, IP19 and IP20.

**Table 2:** Study sample. Abbreviations: f = female, m = male, n = number

Residence in (federal state)		Gender	Licensure status			Immigration	
			temporary	permanent	none	by visa	as refugee
Bavaria	n = 9	m = 9, f = 0	7	1	1	8	1
Berlin	n = 4	m = 4, f = 0	0	2	2	4	0
Schleswig-Holstein	n = 2	m = 1, f = 1	2	0	0	2	0
Rhineland-Palatinate	n = 3	m = 3, f = 0	1	0	2	0	3
North Rhine-Westphalia	n = 2	m = 0, f = 2	0	2	0	2	0
**total**	***n*** **= 20**	**m = 17, f = 3**	**10**	**5**	**5**	**16**	**4**

### Informed consent and confidentiality, ethics approval

The study was approved by the Ethics Committee of the University of Regensburg (16–101-0247). Written consent was obtained from all study participants in accordance with the ethics approval. Participants received no incentive and participated voluntarily. All participants completed a short questionnaire asking for information about socio demographic details.

The interviews were conducted face-to-face between July 2016 and January 2017. The interviewer (Y.A.), a Syrian dentist by training, conducted the interviews in the respective private homes of the participants to ensure a convenient and comfortable environment. Interview language was Arabic. The interviews lasted between 20 and 65 min. The research team discussed regularly whether novel aspects were still coming up in the interviews; after 20 interviews, saturation was reached.

### Analysis

The interviews were audio recorded and transcribed verbatim in Arabic and then translated into English. Before the actual analysis, overarching topics were developed in accordance with the interview guide. Three researchers (J.L., J.S., A.S.) read and coded the interviews independently and identified main topics inductively from the transcribed material. Differences between the coding of the researchers were discussed within the research team until consensus was reached. A thorough and detailed content analysis, based on those topics, followed [25]. The first step of the analysis was the identification of frequently recurring themes [23]. Those themes were clustered and explanations developed in an iterative process within the research team.

## Results

During the interviews, several main barriers and difficulties emerged regarding the process of obtaining the licence to practice medicine and finding employment. In the following sections, we will highlight and explain those main barriers, as experienced and perceived by the Syrian doctors.

### The access to official information about requirements and necessary documents is limited – immigrants rely on their peers and word of mouth

According to the interview partners, the requirements for licensure are complex, and they found it hard to obtain adequate information (regarding the licensure process itself, necessary documents as well as their validation). It appears as though there is no reliable official information source. Many interview partners agreed that it was nearly impossible to receive individual information from the professional regulatory bodies in charge.

-
  *Before I applied for the permanent license, I tried to call them [the authority] several times, and sent them emails, in order to find out which documents are required for the application … but no one answered me. [IP12]*

Sources of information are reported to be mainly other Syrian immigrant doctors who share their knowledge and social media channels, e.g. special Facebook groups that provide information about proceedings and helpful organizations.

-
  *I looked for a way … to be allowed to practice as a doctor in Germany … I realized that in Berlin, it’s not easy to get one’s documents approved, because the doctor is required to have a verifiable working experience of three years minimum. That is what all my friends, who know the administration in Berlin longer than me, told me. One of them told me about an institute that offers [training for] the theoretical exam to get the permanent license … I enrolled in this course, and I passed the exam and obtained the license. [IP09]*
-
  *From the experience of other doctors, and the people who are communicating on the Internet, I knew that the Approbation takes a long time nowadays, so after I got the temporary permit I [immediately] applied for the permanent permit. [IP14]*

### The immigrated doctors are exasperated at the bureaucratic requirements, which are perceived to be partly absurd, ever shifting, and varying between regions

Most of the interviewed persons report some kind of preparation before coming to Germany, e.g. obtaining necessary documents, translations and authentications. Nevertheless, once handed in to a German authority, these documents often prove to be incomplete, inconsistent or do not meet German standards, according to the interview partners. In addition, the interviewed doctors realized that there are differences between the German and the Syrian system in the use and meaning of some necessary documents, e.g. birth certificates or certificate of conduct. Many applicants feel mistreated, as for them a) it is not understandable why those documents are needed in the first place, and b) it is extremely difficult to get hold of the required certificates.

-
  *Where I can get my birth certificate? My city is under the rule of ISIS now, how can they think that I can go there and bring this paper? Here in Germany they don’t know that in Syria, this document is valid for 3 months only. In Syria we have our ID and Passport. [IP15]*
-
  *I didn’t authenticate the documents because some people said: ‘In Germany they don’t accept the translations made in Syria.‘ But how can I have them translated in Germany, and after that send it back to Syria and Lebanon in order to get a validation?! [IP16]*

The federal system of Germany seems to add to the confusion and irritation, as the list of required documents and accomplishments varies among federal states. So does the time span that it takes to receive the temporary and permanent license. The interviewees report that they tried to file their documents in federal states whose requirements are perceived to be easier to accomplish than those in other states. Sometimes, they deliberately choose “unattractive” sites because they hope the process may be quicker here (because of less competition). Differences between federal states are communicated through personal networks of Syrian doctors.

-
  *Here in Germany, there are 16 federal states, and there are 16 rules to get the permanent license. [IP17]*
-
  *I wish that this chaos in Germany would be become organized. The [foreign] doctors seek out a place to have their documents approved, and they are asked for certain documents which are not required in other regions in Germany. I wish they would make clearer regulations in Germany for the documents. I know many doctors who went to [the federal state of] Saxony first to have their documents approved, but as it didn’t work out there, they moved to [the state of] Bavaria instead … searching for an easier way to get it done. [IP16]*

Requirements do not only differ between federal states. It became clear that necessary accomplishments and credentials were being changed over time, which hampers the application process and leads to irritation and frustration, according to the interviews.

-
  *Every day there are new rules, new things that are required. [IP01]*
-
  *… The problems started when I started applying for the temporary license. Some rules changed and the authorities were now requiring a C1 course in medical German to obtain the temporary license! After I started the C1 language course, they required the exam for medical terms. When we completed one step, they asked for another step. [ … ] When I arrived here, I learnt that nothing is stable in Germany. [IP05]*

### The licensure process is perceived as lengthy and completely non-transparent, and the impossibility of inquiries or feedback drives the interview partners to despair

Unlike obtaining the temporary license, which was mostly described as a (relatively) speedy and simple procedure, the application for the permanent license (“Approbation”) takes very long, i.e. several months up to longer than a year, as many interview partners report.

-
  *The processing of my documents was so tiring here in Germany. … My case should get a record in the list of persons waiting for the longest time in German administration. … I applied for the “Approbation” in June 2015 and I got it in July 2016, which is around 14 months later. [IP15]*
-
  *I have been waiting for too long now, for more than 6 month. I don’t know what’s happening! They asked for certain documents, and I sent those in. This is the last thing I know … . My documents should be complete now, but they haven’t called yet to confirm it. [IP01]*

The long and unpredictable waiting time causes psychological distress for the applicants, as they remain in a status of uncertainty for a long time. The interviewees felt that the procedure of assessing their credentials (and the reasons for the long handling time) were not transparent at all. This applies to temporary and – even more so – to the permanent license. During the process of re-certification, the regulatory body does not communicate the individual steps to the applicants, according to the interviewees. It remains unclear to the applicants whether the documents are complete and correct, in which status the process is, and how long it will take to get a final notification. The interviewed doctors reported that questions relating to their documents and the status of the process were ignored, despite countless efforts to get in touch personally, by phone or by e-mail. This results in irritation, fear and despair.

-
  *For months now I haven’t got any news, and I don’t know what is happening … . No one answers you in this authority. I called them more than 10,000 times, but no one ever answered my calls. … Why does it take so long? This is really affecting the psychological condition of the doctors … Why are they not answering my calls or inform me about the stage of processing the documents?! That’s what really makes me feel miserable and sad. [IP03]*
-
  *Don’t try [to reach anyone] by phone, you will never get anyone answering you in the authority office. … I guess I called them for more than 500 times. And if somebody answered you, it was not the person who was processing your documents. … I went to the authority office, [but] I regretted going there because I didn’t get anything from them. The employees were really so annoying, because they always said: ‘Wait and wait’, that is the only thing they can say. [IP15]*
-
  *I kept calling them and sending emails in order to learn more about the status of my documents, but I got nothing back. Even the manager of the hospital tried, but didn’t get any answer. … After two months I tried again to call them and send emails … just the answering machine went ‘You are calling out of working hours’, even though it was within the working hours. [IP12]*

The lack of transparency was linked to the perception of arbitrariness. The interview partners reported to have developed strategies in order to cope with the situation, e.g. trying to avoid certain employees. The perceived power of the administration staff over the Syrian doctors’ fate is described as a psychological burden for the applicants, as they feel they are at the mercy of people and there are no rights that they can rely on. This was true for the decision of employees as well as for the external review.

-
  *The problems result from … the [random decision of the] person who assesses your documents. There are no clear steps. You can have two [Syrian] doctors who studied at the same university, finished their studies in the same year, - and then one of them can obtain the approbation without any exam, and the other one will need to take another exam. At the end of the day, only one person makes the decision about you. The external reviewer studies all the papers and the reports and will then tell you the final decision. And of course we cannot meet this person or talk with them. We don’t even know their name. [IP01]*
-
  *[In the] Government authority office … I told [one employee] that I needed to object to the Reviewer’s decision. [ … ] I saw my contact person by chance. At first he didn’t want to talk to me, but I kept talking and told him: ‘You are the only one who can help me! The external reviewer made a mistake about me, and I need your help!’ He said: ‘If the Reviewer decided something, then that is correct. The Reviewer never makes mistakes. Whatever you will do [to object to that], you’re just wasting your time.’ [IP10]*
-
  *In Germany, people need to be lucky. Each employee does as he or she needs to. The same employee who gave me the permanent license [swiftly] told my husband that he is required to validate his documents. [IP20]*

As the employees were considered the decisive persons in the process of decision, and as they were perceived to differ with regard to requirements, an informal information system developed, according to the interviews.

-
  *There is a page on Facebook for the Syrian doctors in Germany. In it, there are all the names of the employees in the authority written down... the doctors [users] wrote for each employee what documents are usually required by her or him, and commented on the differences between the employees. [IP16]*

Some interview partners explicitly expressed incomprehension for the bureaucracy, the time spans and complications involved. Some also tell episodes of absurdity, such as documents carelessly being lost or going missing in official institutions, or of two institutions simultaneously refusing to hand out documents before the other institution would have acted. They use the words ‘*chaos’*, ‘*farce’,* ‘*not normal’*, ‘*bureaucratic country’,* ‘*big failure’*. The interview partners felt helpless in these situations.

-
  *Lots of Syrian doctors have come to Germany over the last years. So the German government should know by now which documents are available in Syria, and which are not. The number of studying hours for each subject have been the same for more than 20 years now at Syrian universities. But [the German authorities] require each doctor to prove it individually. Why does each single doctor have to prove their working hours again and again? It is such a waste of time and money. [IP04]*

The long time and procedures involved in the licensure process were perceived as disregard for the interview partners’efforts and expertise.

-
  *That is not fair: to work and not being appreciated by anyone for the work you did. I had worked for one and half year in Syria, and here another one and half year … A [Syrian] friend of mine from the hospital was in the same situation … he tried to call [the administration] to ask about the status of his documents, and … the employee told him to call back after a year! He said it ever so simply. One year of your time is nothing to them. [IP13]*
-
  *I am not going to study for another year … I can’t wait any longer to start working. I had worked with patients in Syria for a year and a half. I can’t take a step back and study for another year. [IP06]*

However, some IPs also reported positive encounters with the employees of the authorities, describing them as kind and supportive, “respectful and willing to help” [IP03]. For example, all interviewed women described the interactions with the regulatory bodies as smooth and agreeable.

-
  *My language was so good that when I applied for the approbation, she [the employee] gave me the permanent license directly. It took only 5 weeks for me to get it. In the end you can get it if you convince the employee. [IP19]*

### The job search, especially for internships, is lengthy, but doctors feel treated in a respectful way – if they are not wearing a headscarf

Throughout all interviews, it became clear that the application for internships turned out to be a frustrating experience for the interview partners, as numerous applications were not responded to. Many interview partners have come to believe that it would be more effective to introduce themselves personally in the hospitals instead of writing applications.

-
  *I went to more than ten hospitals, and in each one I went to three departments asking for an internship. It was not easy at all to find a position for an internship of four months. I had started by writing emails … but I did not get any answer from them … Because of that, I went by myself to each hospital and asked. It took me one month to find this internship. [IP06]*

In addition, it is incomprehensible for the interviewed Syrian doctors why applications for internships are turned down, especially if those are unpaid. This was perceived to be especially humiliating as the interview partners used to be experienced doctors in their home country.

-
  *Even the unpaid internship is so difficult to get. I don’t understand why, even when we help for free, they don’t need us. [IP13]*
-
  *It is the worst feeling ever. I used to be a doctor working in a university hospital in Syria, and … not any doctor can do what we did in Syria! During the war we operated on a lot of [patients with] complicated injuries, and then in Germany, if you apply for an unpaid internship to help them, you get the answer ‘No’! That makes me very depressed, and makes me feel like going back to Syria even if it’s war. We have a lot of knowledge, a lot of experience, we just need to enter [the system] and see how the system here works, we need to start practicing in Germany and support them with our knowledge – but in the end they refuse us! [IP09]*

In some stories, an internship was not found before a German acquaintance (doctor, teacher) with some relation to a hospital intervened personally.

-
  *In Germany, personal relationships are the most important thing to get a job. The first working place is always the most difficult. I got my first job through a retired German teacher. She knows the chief consultant in a hospital, and she told him about me. I did an internship first and after that, I got my current job. [IP16]*
-
  *Most of my emails didn’t get an answer, not even a negative answer. … I did not give up … I searched again and again, until I met a … retired doctor. She … helped me a lot, she knew a physician in Berlin, she spoke to him and he offered me two months of training in his clinic. [IP07]*

Most interviewees describe the job interviews (for internships or working positions) they had as fair and friendly. The interview partners emphasized experiences in which they were being addressed in a respectful way, implying that they were seen as professional colleagues.

-
  *In general, the experience of the job search was not so bad for me, at least the employers treated me like a doctor. Even if they turned my application down, they still spoke with me in a really respectable way. [IP07]*

Being treated in an unfriendly way due to their immigration background was especially painful when it happened in a hospital environment, which the interview partners considered a familiar habitat that they were entitled to.

-
  *When I went to the hospitals, … some secretaries talked to me in a very respectful way, and made me feel treated as a doctor. But there were others who didn’t even let me enter the [respective] hospital [department], … talking to me as though they would like to say ‘Get away from here!’ … Are we [Syrian doctors] coming from another world? I’m a medical doctor entering a hospital, what’s the problem? [IP04]*
-
  *I wish the German people ... gave us the chance to show what we can [in the job interviews]. I [would like] them to see how I can talk, and how I can be good with other people. I’m not an alien, I’m a human being like them - I’m just wearing this Hijab. [IP19]*

The interviewed women doctors described that they suspected or confronted difficulties in the application process because they were wearing a Hijab. In job interviews, the fact that the applicant was wearing a Hijab could raise doubts about her competence to treat patients, according to the experiences of all three interviewed female doctors. They reported few cases where the headscarf was named explicitly as a reason not to employ the female applicant.

-
  *When I came to the hospital to take part in the interview, they started talking to me about what I look like, about my integration and my Hijab. … . They didn’t ask me about my expertise or training, just: ‘You’re here now for one year and half, and you’re still wearing your Hijab?’ … That was the worst interview ever, and I was so depressed. … After that I felt so hopeless to find a job here in Germany. [ … ] Later I did another interview, and they did not talk about my Hijab, they just said: ‘We don’t care about religion’ … That was so great for me. [IP08]*
-
  *In the interviews, I realized that most of the people have wrong ideas about the Hijab. They think that if someone wears a Hijab, she’s not a proper doctor. They asked me: ‘Are you allowed to touch the patients?...Isn’t that forbidden in Islam?’ I answered: ‘No, of course not, we have to help the sick people.’ … They told me: ‘You look so conservative and extreme in your religion, we’re afraid that you will not integrate well with the other doctors in the team’. ...After I finished my explanations … they said: ‘You’re more open-minded than we thought’ … , I didn’t benefit from all my accomplishments here [i.e. language training, efforts to get the Approbation], because I am wearing a Hijab. A piece of clothes determines my destination! [IP19]*

As a reaction to this perceived discrimination, the interviewed women developed different strategies: one was trying to avoid including a photograph in written applications, whereas the other deliberately attached a photograph with her wearing a hijab, so if she was invited to the job interview, she could trust that the hijab is not an issue for the potential employers. The third woman had started to wonder if she needed to take off her Hijab, against her will, when she would want to find a job.

-
  *I put my portrait with veil on the CV, so they know that I am wearing it. So when they are willing to do an interview with me, that means they accept what I look like, or that 50 % of all the problems [that can be expected in the interview] are already ruled out, which is the Hijab. [IP08]*
-
  *Very often I felt depressed, and all the time I thought about my Hijab. If I want to find a good job, will I have to take off my Hijab?! Even some of my friends told me: ‘Why are you putting on your Hijab?’ [IP20]*

## Discussion

### Principal findings

The interviews show that upon arrival in Germany, Syrian doctors are highly motivated to use their skills and expertise in clinical patient care. 16/20 interview partners came by visa and had to a certain extent planned and prepared their licensure process before immigration. Once in Germany, they are then confronted with a complex, lengthy, ever-changing and non-transparent application procedure, which is perceived by them as a partly unfair, partly arbitrary bureaucracy. They describe a Kafkaesque system where they feel at the mercy of Government employees and reviewers who ask for absurd and impossible accomplishments, refuse to give information, and act at random. As a consequence, the interviewees report to be depressed, irritated and/or in despair. Their frustration also reflects their loss of social status that they experience after migration. This status is at least partly restored in the job interviews when they feel to be ‘treated like a doctor’ again. All three interviewed female doctors, however, reported that their wearing a Hijab led to episodes of discrimination in their job search. According to the interviews, informational and practical support from official institutions was scarce, and instead the Syrian doctors relied on peers or, in some cases, German friends to understand the requirements and find a job placement.

### Strengths and weaknesses

To our knowledge, this study is the first to shed light on the licensure and application process of Syrian immigrant doctors in Germany. Due to the qualitative design of the study, it is possible to track and understand the situation and challenges that the interviewed doctors find themselves in. The interviewer was a Syrian dentist who was himself in the process of applying for a permanent license, which facilitated not only the recruitment of interview partners, but also helped build mutual trust and understanding between interviewer and interviewees, which is beneficial for having the interview partners open up and speak freely. As the interviewer was in a similar situation as the interviewees, this may have induced a feeling of solidarity or fraternization, but it cannot be entirely ruled out that questions were unconsciously asked in a suggestive formulation, as the interviewer himself was affected by problems in applying for his license. The sample was heterogeneous enough to grasp differences between federal states, the gender of applicants, and their route to Germany, and therefore allows a broad picture with regard to different experiences.

Our study also has some limitations. Qualitative studies do not claim to produce representative data but are meant to explore the range of possible experiences. Nevertheless, we cannot rule out that the recruitment strategy might have predominantly attracted those Syrian doctors who are especially unsatisfied with the licensure process. There was a call for immigrant doctors to participate in an interview about the licensure process, and it is possible that those doctors who had felt treated especially unfair and wanted to complain about their situation were more likely to respond to this call. This possible selection bias may have led to an accumulation of negative accounts. Additional purposive recruitment and snowball sampling may have counterbalanced this potential effect. In addition, we specifically considered deviant statements (e.g. positive and favourable accounts of document processing), and mentioned those in the results section as appropriate (‘deviant case analysis’ according to Mays and Pope [26]).

A further drawback is the small number of female participants. This may reflect the probable proportion of female Syrian doctors residing in Germany as compared to male Syrian doctors [27]; nevertheless, this subsample is too small to allow for understanding the special situation of Syrian women doctors in Germany. It is interesting that all three female interview partners reported comparable experiences about their wearing a Hijab in job interviews.

### Comparison with other studies

Klingler and Marckmann (2016) interviewed foreign doctors employed in German hospitals, mostly from Eastern Europe and Russia. Although the interviews dealt with working conditions and difficulties experienced in the hospital setting, the results also briefly hinted at the licensure process. The interview partners described the licensure process as ‘slow, confusing, and overly bureaucratic’, which is, in a nutshell, reflected in the Syrian doctors’ experiences in our study [17]. Also a Canadian review concluded that foreign health professionals often lack information about the credential verification and reviewing process, and about the programs and resources available to initiate this process [28]. The complexity of licensure procedures is oftentimes underestimated by applicants. International medical graduates starting their career in Norway reported the authorisation process as ‘more cumbersome and time-consuming than they had anticipated’. [19] The qualitative study by Jirovsky et al., investigating migration reasons and experiences of health worker coming to Austria, also showed that the accreditation process is perceived as a major barrier. The majority of the participants had to repeat parts of their training in order to receive the necessary licence. This led to a feeling of being deskilled and to a loss of social status [20].

Klingler and Marckmann also found evidence of rejection and discrimination at the workplace, attributed to the interviewees’ status as ‘foreigner’, and a lack of trust in the immigrant doctors’ professional expertise [17]. We did not focus on the working experiences of the interviewed Syrian doctors, but still the discrimination aspect came up among interviewees when talking about the job application. It was only women, however, who reported being discriminated against, and the rejection was related to their religion, i.e. wearing a hijab. The male interview partners reported to be treated with respect in the job interviews; they experienced rejection and condescension, but mostly by administrative staff in the licensure process, not by professional colleagues. Abu-Ras, Wahiba et al. report similar experiences of female Muslim physicians in the USA [29]. In their study about religious identity and workplace discrimination in the United States, Padela, Aasim et al. could show that Muslim physicians felt under greater scrutiny at work, and also partly felt discriminated against at work, especially the most religious ones [30].

### Implications for policy and practice

Immigrated Syrian doctors represent a potential solution for addressing the shortages of human resources in the German health care system, and also for addressing special needs of the increasing number of patients who have fled Syria over the last years. Therefore, policies and programs that support newly arrived Syrian health workers in their process of professional re-establishment would be of specific importance. The interviews showed that there are various challenges that could be addressed, as all interviewed Syrian doctors struggled to become recertified and employed within their profession.

For example, the study highlights a need to establish an official information source giving accurate details about the licensure process, the required documents, the estimated waiting times, the regulations for courses and exams, and the criteria used for reviewing the credentials. This information source should be easy to understand and to access (e.g. a website), should account for the continuous changes in the regulations and the differences across federal states. A good example are specific settlement programs that have been implemented in Canada. They include specific websites (‘access centers’) which provide information about the licensing process and contain many different resources such as guides, toolkits, and videos (http://www.healthforceontario.ca/en/Home/All_Programs/Access_Centre) [28]. The differences in requirements across federal states led to confusion and feeling of unfairness among the participants, and induced some of them to move from state to state. Therefore, it may be beneficial to harmonize the licensure processes across the different federal states. A good example for this is Australia, which has streamlined the assessment processes for overseas qualified doctors across its states and territories by developing a national assessment process with standardized pathways [31].

It transpired in the interviews that the interviewed doctors would be supported significantly if a personal counselling service could be installed. Telephone hotlines or online counselling could be an approach to address this need, or even special training programs [32]. In Canada, for example, case management and mentorships were implemented with the aim to helping immigrant health workers navigate the licensure and examination processes [28, 33]. The applicability of similar approaches should also be checked in Germany.

The long waiting times and difficulties in reaching administrative staff can probably be attributed to relative staff shortage which has resulted from the sudden increase in applications from Syrian immigrants. Investing resources in facilitating and speeding up the licensure process, e.g. by employing extra staff, could prove beneficial in the longer run, as it allows Arabic speaking doctors to enter the job market more quickly, and thus help fill the vacant positions in many hospitals. It should also be discussed whether the licensure processes can be changed or simplified as to be more efficient; for example, one interview partner pointed out that the curricula of medical programs are identical in all Syrian universities, so maybe the study certificates need not be checked for every applicant individually. Covell et al. report that in Canada, for example, there exist ‘credentialing agencies’ that provide the initial equivalency information for professional regulatory bodies [28]. On the other hand, it would be important to hear the perspective of the administrative staff in the German Chamber of Physicians or in the job centers as well, to obtain a balanced view of the licensure process and the interactions involved. To our knowledge, such a study has not been performed yet.

The interviews also showed that the many immigrated doctors struggled with the job application procedure. Almost all interview partners who had applied for a position reported that their written applications were mostly unsuccessful. We can only speculate as to why the applications were ignored or turned down. There may have been no vacancy at the time of application; responsible hospital managers or heads of clinical departments, who make the decisions about applications, may not have considered the applicants a good fit for the respective vacancy, or may have been concerned about language skills. Prejudice or general concerns about foreign applicants may have also played a role. On the other hand, one could also speculate that the form of applications may not have met German standards. In Syria, doctors do not have to write a formal application, as medical jobs are usually distributed via the Syrian medical syndicate. It could therefore be useful to offer Syrian immigrants help for the application process, which could be part of their cultural competence. The New Zealand Ministry of Business, Innovation and Employment, for example, offers specific courses for migrants in which they role-play job interviews and learn how to talk with confidence about their work experience, skills and their careers [34].

Whereas male interview partners described the job interview situation as fair and respectful, female doctors wearing a headscarf were sometimes challenged by hospital managers’ (or consultants’) worries that their religion could prevent appropriate patient care. This finding implies that there are misunderstandings about the Muslim religion and its potential interference with medical patient care, resulting in insulting remarks and discrimination. These misunderstandings and the consequent unfair treatment should be addressed. The hospital management as well as other healthcare institutions (Chamber of Physicians etc.) should foster a norm of respect and diversity, and thereby create a welcoming atmosphere for Syrian migrant doctors [17]. Specific interventions can improve the cultural competence and diversity management of hospital staff; also Arabic patients could benefit from those interventions [35].

Further research could build on these findings. For example, it may be interesting to further explore the specific situation of female doctors. All women in our sample reported fewer difficulties in the licensure processes, but were challenged in the job interviews because of their headscarves. The female subsample is too small, however, to generalize these findings; a study focusing on Syrian women doctors and their experiences in the licensure and application processes would be helpful in understanding gender-specific needs. In addition, longitudinal studies following up on the experiences of the Syrian doctors may help understand whether or not adverse experiences that were made in the application processes led to permanent distrust in the German medical system, or how integration processes develop over time while working in German hospitals.

## Conclusion

This study could highlight several obstacles that medical doctors from Syria face when applying for a license and a job in Germany. Bureaucratic hurdles, lack of transparency in terms of requirements and processes, and unsympathetic, sometimes discriminatory communications are consistent experiences reported by immigrant doctors. This does not only slow down the process of integrating Syrian doctors into the German health care system, but also leads to severe frustration and desperation among the applicants. The German medical system would benefit from programs and resources that render the application process more efficient and assist foreign doctors in navigating the German bureaucratic system. In addition, hospitals should receive more support for managing cultural and religious diversity, as well as for their efforts to help Syrian immigrant doctors overcoming various barriers.

## Data Availability

The datasets generated and/or analyzed during the current study are not publicly available due to the preservation of anonymity of the interview partners but are available from the corresponding author on reasonable request.

## Abbreviations

F: Female
IP: Interview Partner
M: Male
N: Number
Yr.: Years
